# Regulation of the programmed cell death protein 1/programmed cell death ligand 1 axis in relapsing–remitting multiple sclerosis

**DOI:** 10.1093/braincomms/fcad206

**Published:** 2023-07-25

**Authors:** Thanos Tsaktanis, Mathias Linnerbauer, Lena Lößlein, Daniel Farrenkopf, Oliver Vandrey, Anne Peter, Ana Cirac, Tobias Beyer, Lucy Nirschl, Verena Grummel, Mark Mühlau, Matthias Bussas, Bernhard Hemmer, Francisco J Quintana, Veit Rothhammer

**Affiliations:** Department of Neurology, Klinikum rechts der Isar, Technische Universität München, Munich 81675, Germany; Department of Neurology, University Hospital Erlangen, Friedrich-Alexander University Erlangen-Nuernberg, Erlangen 91054, Germany; Department of Neurology, Klinikum rechts der Isar, Technische Universität München, Munich 81675, Germany; Department of Neurology, University Hospital Erlangen, Friedrich-Alexander University Erlangen-Nuernberg, Erlangen 91054, Germany; Department of Neurology, University Hospital Erlangen, Friedrich-Alexander University Erlangen-Nuernberg, Erlangen 91054, Germany; Department of Neurology, University Hospital Erlangen, Friedrich-Alexander University Erlangen-Nuernberg, Erlangen 91054, Germany; Department of Neurology, University Hospital Erlangen, Friedrich-Alexander University Erlangen-Nuernberg, Erlangen 91054, Germany; Department of Neurology, University Hospital Erlangen, Friedrich-Alexander University Erlangen-Nuernberg, Erlangen 91054, Germany; Department of Neurology, Klinikum rechts der Isar, Technische Universität München, Munich 81675, Germany; Department of Neurology, Klinikum rechts der Isar, Technische Universität München, Munich 81675, Germany; Department of Neurology, Klinikum rechts der Isar, Technische Universität München, Munich 81675, Germany; Department of Neurology, Klinikum rechts der Isar, Technische Universität München, Munich 81675, Germany; Department of Neurology, Klinikum rechts der Isar, Technische Universität München, Munich 81675, Germany; Department of Neurology, Klinikum rechts der Isar, Technische Universität München, Munich 81675, Germany; Department of Neurology, Klinikum rechts der Isar, Technische Universität München, Munich 81675, Germany; Munich Cluster for Systems Neurology (SyNergy), Munich 81377, Germany; Ann Romney Center for Neurologic Diseases, Brigham and Women’s Hospital, Harvard Medical School, Boston, MA 02115, USA; Eli and Edythe L Broad Institute of MIT and Harvard, Cambridge, MA 02142, USA; Department of Neurology, Klinikum rechts der Isar, Technische Universität München, Munich 81675, Germany; Department of Neurology, University Hospital Erlangen, Friedrich-Alexander University Erlangen-Nuernberg, Erlangen 91054, Germany

**Keywords:** neuroinflammation, autoimmunity, checkpoint, co-regulation, PBMCs

## Abstract

The programmed cell death protein 1/programmed cell death ligand 1 axis plays an important role in the adaptive immune system and has influence on neoplastic and inflammatory diseases, while its role in multiple sclerosis is unclear. Here, we aimed to analyse expression patterns of programmed cell death protein 1 and programmed cell death ligand 1 on peripheral blood mononuclear cells and their soluble variants in multiple sclerosis patients and controls, to determine their correlation with clinical disability and disease activity. In a cross-sectional study, we performed in-depth flow cytometric immunophenotyping of peripheral blood mononuclear cells and analysed soluble programmed cell death protein 1 and programmed cell death ligand 1 serum levels in patients with relapsing–remitting multiple sclerosis and controls. In comparison to control subjects, relapsing–remitting multiple sclerosis patients displayed distinct cellular programmed cell death protein 1/programmed cell death ligand 1 expression patterns in immune cell subsets and increased soluble programmed cell death ligand 1 levels, which correlated with clinical measures of disability and MRI activity over time. This study extends our knowledge of how programmed cell death protein 1 and programmed cell death ligand 1 are expressed in the membranes of patients with relapsing–remitting multiple sclerosis and describes for the first time the elevation of soluble programmed cell death ligand 1 in the blood of multiple sclerosis patients. The distinct expression pattern of membrane-bound programmed cell death protein 1 and programmed cell death ligand 1 and the correlation between soluble programmed cell death ligand 1, membrane-bound programmed cell death ligand 1, disease and clinical factors may offer therapeutic potential in the setting of multiple sclerosis and might improve future diagnosis and clinical decision-making.

## Teaser

Tsaktanis *et al*. reveal distinct expression patterns of membrane-bound and soluble programmed cell death ligand 1 in relapsing–remitting multiple sclerosis, correlating with disease severity. These findings demonstrate regulation of programmed cell death protein 1/programmed cell death ligand 1 signalling and emphasize its potential as a disease surveillance and therapeutic target.

## Graphical Abstract

**Graphical Abstract:**
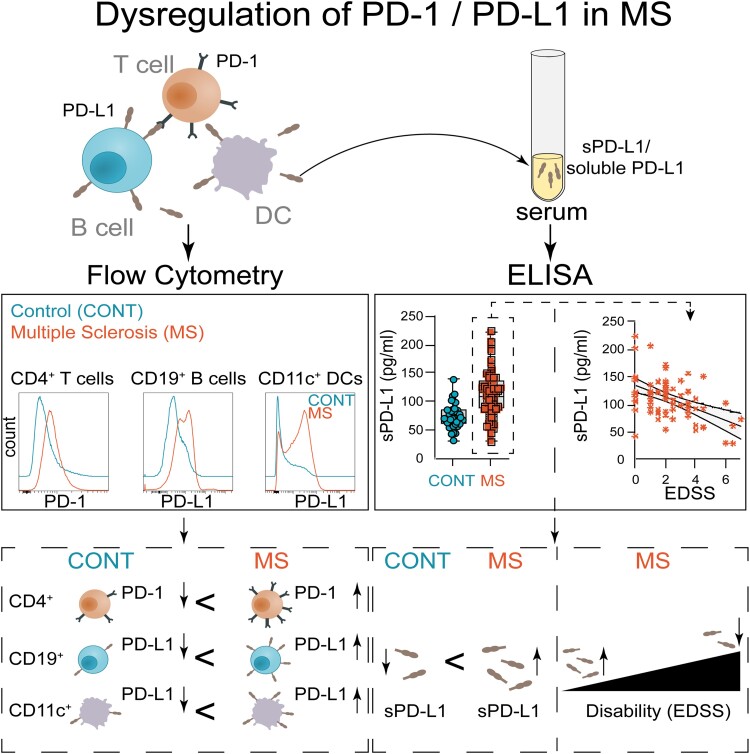

## Introduction

Multiple sclerosis (MS) is a chronic inflammatory, autoimmune demyelinating disease of the central nervous system (CNS) with ∼85% of the patients presenting with relapsing–remitting symptoms (RRMS).^1^ While research has brought forward multiple disease-modifying therapies (DMTs) that reduce relapse rate and disability in patients with RRMS, there are limited therapeutic options for progressive stages of MS.^2-5^ Despite recent studies suggesting that establishing highly efficient therapeutics early in the RRMS stage might delay conversion into progressive disease,^4,5^ these treatments are associated with potential severe side effects. Thus, selecting the appropriate therapy for each patient based on clinical and paraclinical parameters is of utmost relevance.

In MS, autoreactive T cells are primed by their interaction with antigen-presenting cells (APCs) in the peripheral immune compartment and migrate into the CNS, where they are reactivated and consequently drive pro-inflammatory and neurotoxic processes, which lead to tissue damage and neurological deficits.^6-8^ In this context, co-stimulatory and co-inhibitory signalling are essential to regulate the immunological response of autoreactive immune cells.^9,10^ Among other co-inhibitory pathways, the interaction between programmed cell death 1 ligand 1 (PD-L1) and its cognate receptor PD-1 has gained increasing attention in the context of neoplastic diseases but also in autoimmunity, where it modulates inflammation in peripheral immune cells and the CNS.

In addition to their membrane-bound forms, PD-L1 and PD-1 also exist in soluble variants (sPD-L1 and sPD-1) shedded from cellular surfaces.^11,12^ Even though their function is not fully understood, sPD-L1 levels in sera of oncology patients correspond with disease progression and prognosis.^13-15^ Correspondingly, it has been demonstrated that soluble factors in blood plasma exert significant influence on both innate and adaptive immune responses.^16-20^ Indeed, recent research demonstrates that soluble variants of co-inhibitory molecules such as PD-L1 have the capacity to prevent T cell activation in direct *in vitro* assays.^21-24^ Functional aspects of these soluble variants of immune checkpoint molecules in autoimmune disorders remain to be investigated, but several examples of their relevance have been demonstrated in systemic autoimmune diseases like autoimmune hepatitis or systemic sclerosis, where sPD-1 and sPD-L1 serum levels and membrane-bound PD-1 (mPD-1) or PD-L1 (mPD-L1) on T and antigen-presenting cells are linked to disease activity and treatment response.^25-27^

Conversely, ground-breaking therapeutic strategies in oncology inhibiting the PD-1/PD-L1 axis have been associated with CNS demyelination and even MS onset as potential side effects, suggesting the relevance of this axis on CNS pathology via direct and indirect mechanisms.^28^ While the modulation of PD-1/PD-L1 signalling in the context of MS may offer substantial potential for the development of targeted therapeutic approaches, we are still lacking fundamental knowledge on how PD-1/PD-L1 is regulated by distinct immune cell subsets and to what extent it correlates with disease activity and disability measures in MS.

In this study, we examine the expression of membrane-bound PD-L1 and PD-1 on peripheral blood mononuclear cells (PBMCs), as well as their soluble forms sPD-L1 and sPD-1 in the sera of RRMS patients and controls. We define distinct cellular PD-1/PD-L1 expression patterns and investigate how serum levels of soluble PD-L1 and PD-1 are altered in respect to measures of disability and disease activity.

## Materials and methods

### Samples

PBMCs were obtained from patients with non-inflammatory diseases (controls, *n* = 27) and relapsing–remitting multiple sclerosis (RRMS, *n* = 49). Blood for longitudinal assessment was collected from 3 RRMS patients with stable clinical and paraclinical disease course at two different time points, 2, 124 and 176 days apart, respectively, and PBMCs were obtained as outlined in the protocol below. Soluble levels of sPD-1 and sPD-L1 were analysed in patients with non-inflammatory diseases (controls, *n* = 36) and RRMS (*n* = 84). The control group suffered from pseudotumour cerebri or primary headache. All serum samples were collected and stored at −80°C using a standardized protocol.^29^ PBMCs were processed by using a Ficoll gradient protocol and then stored in liquid nitrogen.^30^ Viability rates of > 80% were achieved, and regular quality checks of frozen PBMCS in comparison to fresh PBMCs were performed.^30,31^

This study was approved by the standing ethical committee (14/18S) at Technical University Munich.

### MRI

All brain images were obtained using the same 3T Philips scanner as described before.^29^ The scanning process involved two sequences: a 3D gradient echo T_1_-weighted sequence and a 3D fluid-attenuated inversion recovery sequence. For the T_1_-weighted sequence, the orientation was sagittal with 170 consecutive slices of 1 mm thickness. The field of view was 240 × 240 mm, and the voxel size was 1.0 × 1.0 mm. The repetition time (TR) was 9 ms, and the echo time (TE) was 4 ms. As for the fluid-attenuated inversion recovery sequence, the orientation was axial with 144 consecutive slices of 1.5 mm thickness. The field of view was 230 × 185 mm, and the voxel size was 1.0 × 1.5 mm. The parameters for this sequence were a TR of 10 000 milliseconds, a TE of 140 ms and an inversion time of 2750 ms. For those 46 patients, for whom longitudinal MRI data were available 1 year (1; 1) before blood sampling, at blood sampling and 1 year (1; 1) after blood sampling, lesion load, lesion load volume and white matter volume (WMV) were analysed using the software package SPM and its toolboxes CAT12 and LST.^32^ MRI activity was defined as the appearance of new T_2_ hyperintense and/or new gadolinium enhancing lesions on a follow-up MRI.

### Flow cytometry analysis of PBMCs

For the flow cytometric analysis of PBMCs, frozen PBMCs were thawed and rested at 37° in medium, followed by centrifugation at 300 g for 10 min. PBMCs were resuspended in FACS buffer containing phosphate-buffered saline (PBS) (PAA, Pasching, Austria) with 2% foetal calf serum (FCS) (Invitrogen, Darmstadt, Germany). PBMCs were subsequently stained for 30 min at 4 °C, washed and acquired on a CytoFLEX s (Beckman Coulter Cyan, Brea, CA, USA) or Cytek Northern Lights (Cytek Biosciences, Fremont, CA, USA).

The following antibodies were used for staining: CD197 FITC (Biolegend; clone G043H7), CD3 PerCP (Biolegend; clone HIT3a), PD-1 APC (Biolegend; clone A17188B), CD127 AF700 (Biolegend; clone A019D5), CD45RA APC-Cy7 (Biolegend; clone HI100)), CD25 BV421 (Biolegend; clone BC96), CD4 BV510 (Biolegend; clone SK3), CD196 BV650 (Biolegend; clone G034E3), CD194 PE (Biolegend; clone L291H4), HLA-DR PC5.5 (lifetechnologie LN3), CD183 PE-Cy7 (Biolegend; clone G025H7), CD 24 FITC (Biolegend; clone ML5), CD16 PerCP-Cy5.5 (Biolegend; clone 3G8), CD38 AF700 (BD Pharmingen clone HIT2), CD20 APC-Cy7 (BD L27), CD3 PB450 (BD Bioscience HIT3a), IgD BV510 (Biolegend; clone IA6-2), CD14 BV605 (Biolegend; clone 63D3), CD19 BV650 (Biolegend; clone HIB19), CD56 PE (Biolegend; clone 5.1H11), CD27 ECD (Beckmann; clone 1A4CD27), PD-L1 PE-Cy7 (Biolegend; clone MIH3), LIN (BD CD3 clone SK7, CD14, clone MφP9, CD16 clone 3G8, CD19 clone SJ25C1, CD20 clone L27 and CD56 NCAM16.2), FITC, HLA-DR (PerCP/BD L243), CD11c APC (BD Bly6), PD-1 Super Bright 436 (Invitrogen Clone eBioJ105) and CD123 PE (BD 9F5).

### Statistical analysis

Statistical analyses were performed with Prism software (GraphPad), using the statistical tests indicated in the individual figure legends. No samples were excluded. The investigators were blinded as to sample cohorts when performing soluble PD-1 and PD-L1 enzyme-linked immunosorbent assay (ELISA) measurement, and samples were run in technical duplicates. *P*-values of <0.05 were considered significant. Inter-group comparison was performed using one-way ANOVA. Among parameters with relevant inter-group differences (*P* < 0.1), we computed *post hoc* test Student’s *t*-test. All error bars represent SEM.

### Gating strategy for PBMCs

#### T cell panel

Following live, singlet cell gating (Live–Dead^−^), the following populations were gated: (i) T cells (CD3^+^), (ii) CD4+ T cells (CD3^+^CD4^+^), (iii) CD4− T cells (CD3^+^CD4^−^), (iv) naïve CD4+ T cells (CD3^+^CD4^+^CD45RA^+^CCR6^−^CCR4^−^CXCR3^−^), (v) central memory CD4+ T cells (CD3^+^CD4^+^CD45RA^−^), (vi) Th1 (CD3^+^CD4^+^CD45RA^+^CXCR3^+^CCR6^−^), (vii) Th2 (CD3^+^CD4^+^CD45RA^+^CXCR3^−^CCR6^−^), (viii) Th17 (CD3^+^CD4^+^CD45RA^+^CXCR3^−^CCR6^+^), (ix) memory Th1 (CD3^+^CD4^+^CD45RA^−^CXCR3^+^CCR6^−^), (x) memory Th2 (CD3^+^CD4^+^CD45RA^−^CXCR3^−^CCR6^−^), (xi) memory Th17 (CD3^+^CD4^+^CD45RA^−^CXCR3^−^CCR6^+^), (xii) Treg (CD3^+^CD4^+^CD25^+^CD127^low^), (xiii) naïve CD4− T cells (CD3^+^CD4^−^CD45RA^+^), (xiv) Tc0 (CD3^+^CD4^−^CD45RA^+^CCR6^−^CCR4^−^CXCR3^−^), (xv) memory CD4− T cells (CD3^+^CD4^−^CD45RA^−^), (xvi) Tc1 (CD3^+^CD4^−^CD45RA^+^CXCR3^+^CCR6^−^), (xvii) Tc2 (CD3^+^CD4^−^CD45RA^+^CXCR3^−^CCR6^−^), (xviii) Tc17 (CD3^+^CD4^−^CD45RA^+^CXCR3^−^CCR6^+^), (xix) memory Tc1 (CD3^+^CD4^−^CD45RA^−^CXCR3^+^CCR6^−^), (xx) memory Tc2 (CD3^+^CD4^−^CD45RA^−^CXCR3^−^CCR6^−^), (xxi) memory Tc17 (CD3^+^CD4^−^CD45RA^−^CXCR3^−^CCR6^+^) and (xxii) HLA-DR as suggested in Maecker et al.^33^ A detailed gating strategy is provided in Supplementary Fig. 2.

#### B cell panel

Following live, singlet cell gating (Live–Dead^−^), the following populations were gated: (i) B cells (CD3^−^CD19^+^), (ii) naïve B cells (CD3^−^CD19^+^CD27^−^), (iii) memory B cells (CD3^−^CD19^+^CD27^+^), (iv) transitional B cells (CD3^−^CD19^+^CD24^+^CD38^+^), (v) plasmablasts (CD3^−^CD19^+^CD27^+^CD20^−^CD38^+^), (vi) IgD + memory B cells (CD3^−^CD19^+^CD27^+^IgD^+^), (vii) IgD− memory B cells (CD3^−^CD19^+^CD27^+^IgD^−^), (viii) monocytes (CD3^−^CD19^−^CD14^+^), (ix) classical monocytes (CD3^−^CD19^−^CD14^+^CD16^−^), (x) non-classical monocytes (CD3^−^CD19^−^CD14^+^CD16^+^), (xi) NK cells (CD3^−^CD19^−^CD14^−^CD16^+^), (xii) CD56+ NK cells (CD3^−^CD19^−^CD14^−^CD16^+^CD56^+^) and (xiii) CD56− NK cells (CD3^−^CD19^−^CD14^−^CD16^+^CD56^−^) as suggested in Maecker et al.^33^ A detailed gating strategy is provided in Supplementary Fig. 2.

#### Dendritic cell panel

Following live, singlet cell gating (Live–Dead^−^), the following populations were gated: (i) dendritic cells (Lin^−^HLA-DR^+^), (ii) myeloid dendritic cells (Lin^−^HLA-DR+CD11c^+^) and (iii) plasmacytoid dendritic cells (Lin^−^HLA-DR+CD123^+^) as suggested in Maecker et al.^33^ A detailed gating strategy is provided in Supplementary Fig. 2.

### Analysis of multiparametric flow cytometry data using the OMIQ platform

FCS files were exported for analysis in OMIQ. Following scaling, samples were downsampled to 100 000 live cells and either subjected to manual gating or unsupervised analysis. opt-SNE analysis was performed with 560 iterations (perplexity = 30, theta = 0.5), followed by consensus metaclustering (*k* = 20) using FlowSOM.^34-36^ Heatmaps were generated with OMIQ after Euclidean clustering of mean fluorescence intensities (MFIs). Significance analysis of microarrays (SAM) analysis of PBMCs was performed according to patient group (MS versus CONT) on CD3+CD4 + and CD3+CD4− cells using two-class unpaired testing with 100 permutations (FDR cutoff = 0.1). SAM analysis on mouse cells was performed according to treatment group (PD-L1 versus vehicle) using two-class unpaired testing with 100 permutations [false discovery rate (FDR) cutoff = 0.1].

### Quantitative determination of sPD-L1 and sPD-1 in serum by ELISA

A human sPD-L1 Kit (DB7H10, R&D Systems) was used for the quantitative determination of sPD-L1 levels. Results are reported in pg/ml.

A human sPD-1 Kit (DPD10 R&D Systems) was used for the quantitative determination of sPD-1 levels. Results are reported in pg/ml.

## Results

### mPD-1 is differentially regulated on PBMCs of MS patients

Multiple studies have proposed the use of flow cytometric immunophenotyping of PBMCs and whole blood to establish cytometry profiles for the characterization and clinical management of MS.^37,38^ While most of these studies identify compositional changes in major cellular populations such as B or T cells, only few have focused on subtype-specific surface marker expression.

**Figure 1:**
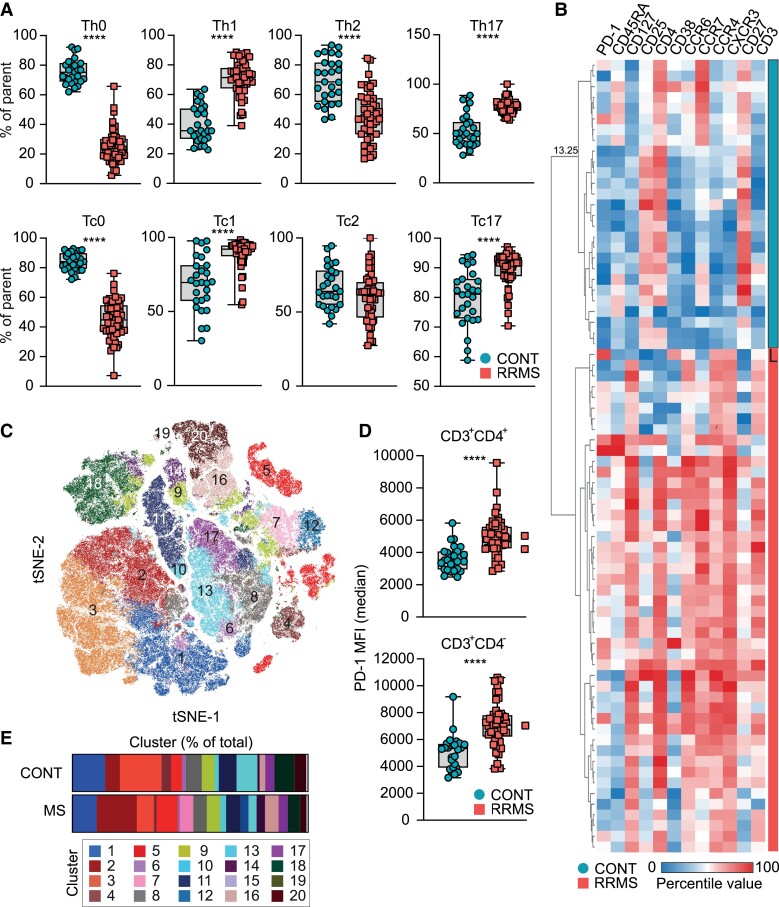
**Multiparametric immunophenotyping of peripheral T cells.** (**A**) Frequencies of Th0 (CD3+CD4+CD45RA+CCR6-CCR4-CXCR3-), Th1 (CD3+CD4+CD45RA-CCR4-CCR6-CXCR3+), Th2 (CD3+CD4+CD45RA-CCR6-CCR4+CXCR3-) and Th17 (CD3+CD4+CD45RA-CCR6+CCR4+CXCR3+) cells in controls (CONT; *n* = 27) and MS (*n* = 49) patients. Unpaired two-tailed *t*-test with Welch’s correction; ****P < 0.0001. (**B**) Hierarchical clustering by Euclidean distance. Clustering was performed based on the expression of CD3, CD4, CXCR3, CD45RA, CD127, CCR7, CD25, CCR6, PD-1, CCR4, CD38 and CD27. Compared were MS (*n* = 49) patients and healthy control (CONT; *n* = 27). (**C**) Unsupervised tSNE clustering of healthy controls (CONT; *n* = 27) and MS (*n* = 49) patients followed by FlowSOM clustering (*k* = 20). (**D**) Median fluorescent intensity of PD-1 in CD3+CD4+and CD3+CD4− cells in healthy controls (CONT; *n* = 27) and MS (*n* = 49) patients. Unpaired two-tailed *t*-test with Welch’s correction; ****P < 0.0001. (**E**) Abundance of clusters identified by FlowSOM clustering in healthy controls (CONT; *n* = 27) and MS (*n* = 49) patients.

Due to the underlying cellular heterogeneity, we aimed to identify specific surface proteins that differ in their expression by distinct T cell subsets and are of potential relevance in the pathogenesis of MS. To this end, we performed multiparametric immunophenotyping of peripheral T cells obtained from patients with RRMS compared to patients diagnosed with non-inflammatory disease, subsequently referred to as controls/CONT [Table 1; Supplementary Table 1, CONT (controls); and Supplementary Table 2, RRMS]. MS patients were characterized by increased frequencies of Th1, Th17, Tc1 and Tc17 cell subsets, while Th0, Tc0 and Th2 cells were reduced (Fig. 1A). This was supported by Euclidian distance clustering, which separated controls and MS patients based on their expression of markers associated with pathogenic or protective T cell subsets (Fig. 1B). Furthermore, dimensionality reduction followed by FlowSOM clustering identified multiple clusters based on their expression of CD4, CD45RA, CCR6, CCR4 and CXCR3, which were expanded or reduced in MS patients compared to controls (Fig. 1C and E and Supplementary Fig. 1A–C). For instance, cluster 12 and cluster 07 were upregulated in MS patients with a fold change of 21.527 and 54.046, respectively (Fig. 1E), and associated with a Th17 phenotype (Supplementary Fig. 1C), while cluster 03 was reduced in MS patients and associated with a Th0 phenotype (Fig. 1E and Supplementary Fig. 1C). Next, we analysed CD3+CD4+ and CD3+CD4− cells using SAM, a statistical method used to identify features from input data described by a response variable, to determine previously described T cell activation markers that substantially differed between MS patients and controls. SAM identified PD-1 as the most significant feature regulated between MS patients and controls (Fig. 1E and Supplementary Fig. 1D and E). Collectively, these data confirm previous reports of an altered T cell subset distribution in MS patients and identify PD-1 as significantly upregulated surface activation marker in MS patients.

**Table 1:** Control and RRMS patients used for the analysis of membrane-bound and soluble PD-1/ PD-L1

Figure	Cohorts	PBMCs	Serum	Females	Age	Disease duration	EDSS	Treatment
	Controls (36)	27	36	25 (69.40%)	33.8 [23.0: 41.5]	None	None	None
1, 2 and 3B and Suppl. 1 and 3	Controls (27)	27	27	23 (85.20%)	34.9 [26.5: 40.0]	None	None	None
3 and Suppl. 4A	Controls (36)		36	25 (69.40)	33.8 [23.0: 41.5]	None	None	None
	RRMS (84)	49	84	31 (64.6%)	42.7 [36.0: 50.0]	8.1 [3.0: 12.0]	2.2 [1.0: 3.5]	65 (77.3%)
1, 2 and 3B and Suppl. 1 and 3	RRMS (49)	49	49	32 (65.3%)	44.2 [37.0: 51]	9.6 [3.0: 13.0]	2.9 [2.0: 4.0]	37 (75.50)
3 and 4 and Suppl. 4B, C and D	RRMS (84)		84	31 (64.60)	42.7 [36.0: 50.0]	8.1 [3.0: 12.0]	2.2 [1.0: 3.5]	65 (77.3%)

‘PBMCs’ indicates the absolute number in the group with available peripheral blood mononuclear cells. ‘Serum’ indicates the absolute number in the group with available sera. ‘Females’ denotes both the absolute count and percentage of females within the group. ‘Age’, ‘Disease duration’ and ‘EDSS’ are presented as mean values, with the 25th and 75th percentiles indicated in square brackets. ‘Treatment’ represents the absolute count and percentage of patients who received treatment, while specific individual treatments, treatment history as well as MRI and clinical activity are listed in Supplementary Tables 1 and 2. No additional noteworthy comorbidities or pharmaceutical treatments were reported among the patients or controls. The diagnosis of MS was determined using established criteria at the time of diagnosis. Note: Suppl, Supplementary.

### mPD-1 and mPD-L1 distinguish MS patients from controls

**Figure 2:**
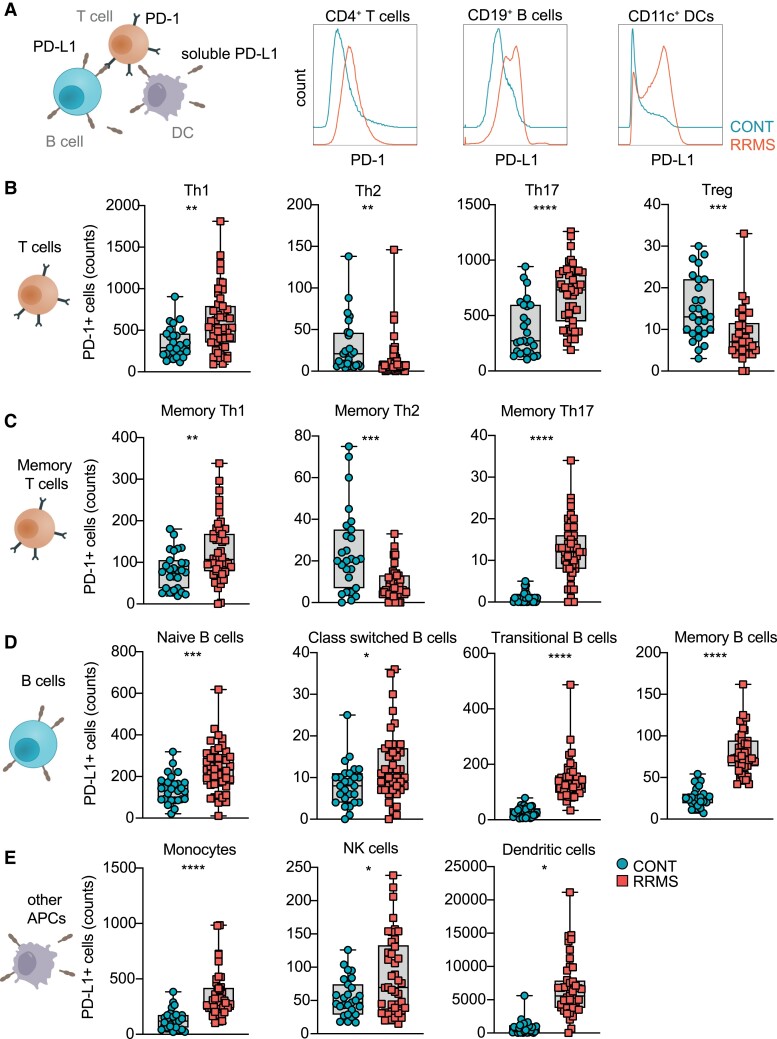
**Membranous PD-1 and PD-L1 expression on PBMCs.** (**A**) Simplified model of PD-1 and PD-L1 interaction between T cells, B cells and APCs. (**B**) Counts of PD-1+ Th1, Th2 and Treg cells in controls (CONT; *n* = 27) and MS (*n* = 49) patients. Unpaired two-tailed *t*-test with Welch’s correction; **P < 0.01, ***P < 0.001 and ****P < 0.0001; Th, T helper cells; Treg, T regulatory cells. (**C**) Counts of PD-1+memory Th1, Th2 and Th17 cells in controls (CONT; *n* = 27) and MS (*n* = 49) patients. Unpaired two-tailed *t*-test with Welch’s correction; **P < 0.01, ***P < 0.001 and ****P < 0.0001; Th, T helper cells. (**D**) Counts of PD-L1+ B cells, class-switched B cells, transitional B cells and memory B cells in controls (CONT; *n* = 27) and MS (*n* = 49) patients. Unpaired two-tailed *t*-test with Welch’s correction; *P < 0.05, ***P < 0.001 and ****P < 0.0001. (**E**) Counts of PD-L1 + monocytes, NK cells and dendritic cells in controls (CONT; *n* = 27) and MS (*n* = 49) patients. Unpaired two-tailed *t*-test with Welch’s correction; *P < 0.05 and ****P < 0.0001; NK, natural killer cells.

To further investigate the role of PD-1 and its cognate ligand PD-L1 in MS patients, we quantified mPD-1 on T cell subsets, as well mPD-L1 on APCs (B cells, dendritic cells and monocytes) and NK cells (Fig. 2A), in PBMCs obtained from RRMS patients and controls using a standardized immunophenotyping procedure (Fig. 2, Supplementary Fig. 2, Table 1 and Supplementary Tables 1 and 2).

We detected an increase in PD-1^+^ pro-inflammatory T cell subsets, whereas PD-1^+^ regulatory T cells were reduced in MS patients as compared to controls (Fig. 2B and C and Supplementary Fig. 3A). Notably, PD-1 expression in Th cells was not influenced by treatment (Supplementary Fig. 3B). In order to determine stability of these signatures in patients with no evidence of disease activity, we investigated the relative expression of PD-1 and PD-L1 on lymphocytes in RRMS patients with stable disease over the course of sequential blood collections and observed stable expression profiles without relevant alteration over time (Supplementary Fig. 3C). Furthermore, we detected an increase in PD-L1^+^ naive, class-switched, transitional and memory B cells in MS patients (Fig. 2D), as well as an increase in PD-L1^+^ monocytes, NK cells and dendritic cells (Fig. 2E). Conversely, PD-1 expression was increased on transitional and memory B cells, as well as on dendritic cells, while there was no significant expression difference of PD-1 in naïve and class-switched B cells, monocytes and NK cells (Supplementary Fig. 3D and E). Collectively, these data demonstrate a differential regulation of the PD-1/PD-L1 axis on cellular subsets of the T cell and APC compartment in patients with MS compared to controls.

### Serum sPD-L1 levels in RRMS patients correlate with disability

**Figure 3:**
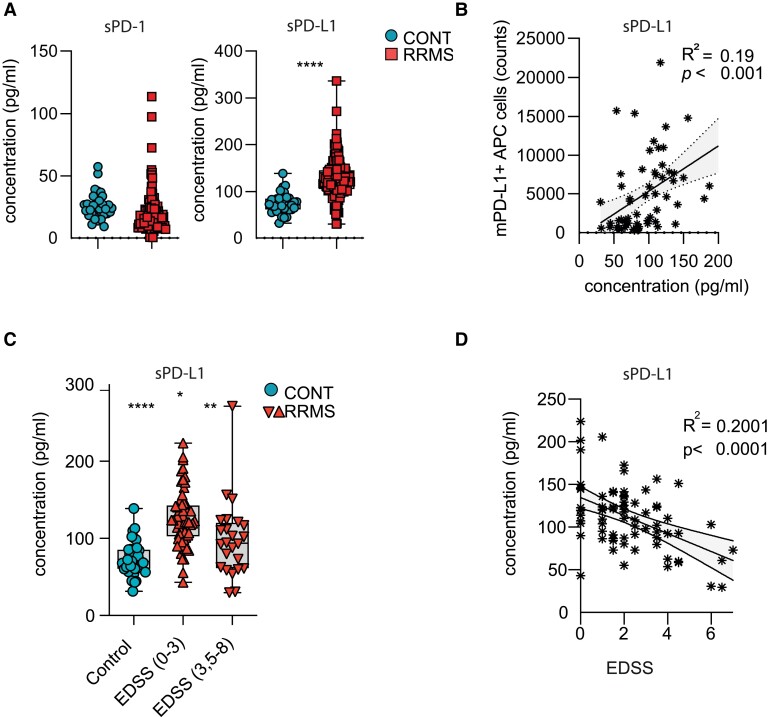
**Correlation of sPD-L1 concentration with disease severity in RRMS patients.** (**A**) sPD-1 (left) and sPD-L1 (right) concentration was assessed in serum samples of patients with RRMS (*n* = 84) and controls (*n* = 36). Significance levels were determined by unpaired two-tailed *t*-test with Welch’s correction; ns, not significant; ****P < 0.0001. (**B**) Linear regression analysis of correlation between soluble PD-L1 concentration in serum and surface expression of PD-L1 (counts) on APCs (B cells, monocytes and dendritic cells) in the cohort where PBMCs and serum were in RRMS (with exclusion of IFN-β-treated patients; *n* = 41) and CONT (*n* = 27) patients available (also see Table 1 and Supplementary Tables 1 and 2). (**C**) sPD-L1 concentration was assessed in serum samples of patients with RRMS patients with exclusion of IFN-β-treated patients (*n* = 70) with different disability levels as determined by EDSS, where higher scores indicate increased disease severity. Significance levels were determined by unpaired two-tailed *t*-test with Welch’s correction; ***P < 0.001. (**D**) Linear regression analysis of correlation between sPD-L1 concentration and EDSS in RRMS patients with exclusion of IFN-β-treated patients (RRMS; *n* = 70). Numbers indicate *R*^2^ and *P*-value of linear regression analysis.

Since both PD-1 and PD-L1 also exist in soluble forms shedded from the cellular surface by matrix metalloproteases (MMPs), we aimed to investigate serum levels of sPD-1 and sPD-L1 in MS patients (Fig. 3A). To that end, we measured sPD-L1 and sPD-1 in sera of 84 patients with RRMS as well as 36 controls (Table 1 and Supplementary Tables 1 and 2) by ELISA. While sPD-1 levels were unaltered between the groups (Fig. 3A, left), sPD-L1 was increased in MS patients as compared to controls (Fig. 3A, right). Moreover, the sPD-L1 concentration was not influenced by age, sex and disease duration (Supplementary Fig. 4A–C), but a stratification by immunomodulatory therapies revealed an increase of sPD-L1 in patients treated with interferon-β (IFN-β) (Supplementary Fig. 4D). Indeed, transcriptional control of PD-L1 by type I interferons has been described before,^39^ highlighting the importance of IFN-β as a driver of PD-L1 also on a soluble level. We thus excluded IFN-β-treated patients from further analyses to avoid confounding of the data set by this variable. Together, these data suggest that in addition to its membrane-bound form, serum levels of sPD-L1, but not sPD-1, are increased in RRMS patients.

Finally, to evaluate whether levels of sPD-L1 correspond to their surface expression on PBMCs, we correlated levels of mPD-L1 on APCs with serum levels of sPD-L1. Indeed, we observed a positive correlation between soluble and membrane-bound forms of PD-L1, suggesting that soluble PD-L1 may be the product of shedding of membrane-bound PD-L1 on APCs (Fig. 3B).

Next, we hypothesized that sPD-L1 levels may correlate with clinical disability measures and analysed sPD-L1 levels in the RRMS cohort in respect to the Expanded Disability Status Scale (EDSS), where higher scores indicate increased disability, irrespective of its potential inflammatory or degenerative nature. We dichotomized EDSS scores into relatively mild clinical impairment (EDSS 0–3) and more severe disability including walking impairment (EDSS 3.5–8). Patients with lower EDSS demonstrated increased sPD-L1 levels compared to patients with higher EDSS (Fig. 3C). Indeed, EDSS scores negatively correlated with sPD-L1 in linear regression analyses (Fig. 3D, Table 1 and Supplementary Table 2), suggesting that increased sPD-L1 levels may be associated with lower disability measures.

### Serum sPD-L1 levels in RRMS patients correlate with MRI activity

**Figure 4:**
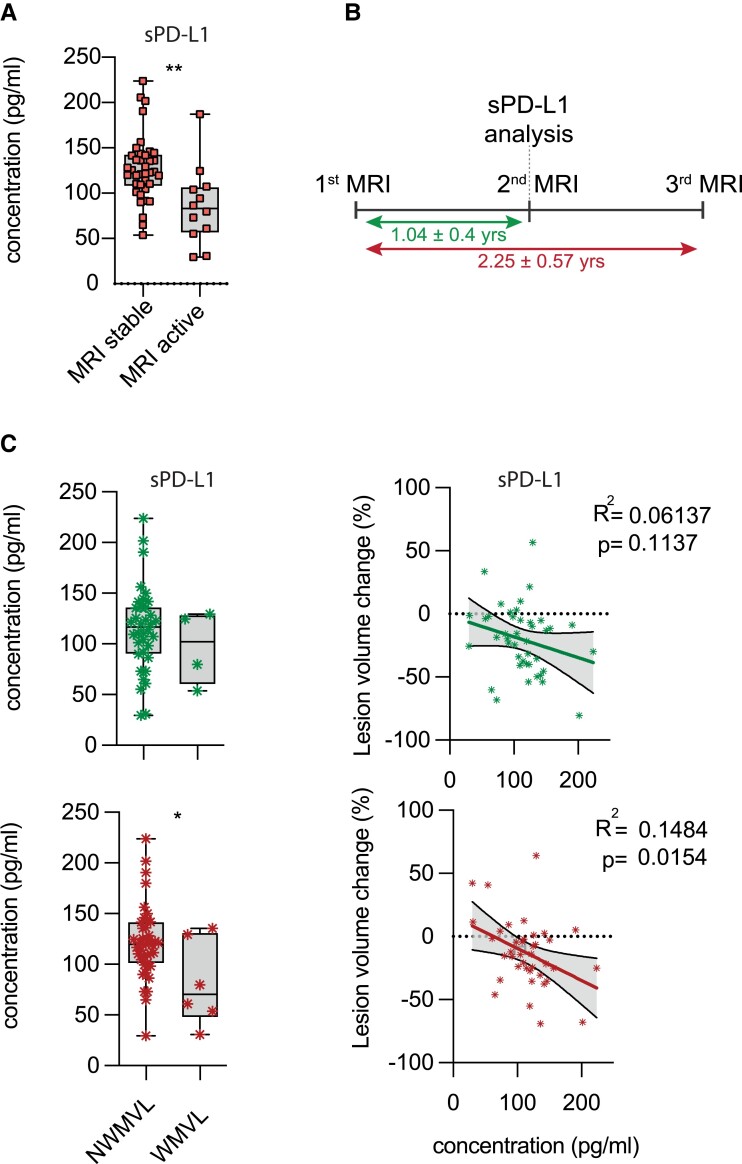
**Correlation of sPD-L1 concentration with MRI activity in RRMS patients.** (**A**) sPD-L1 serum levels (pg/ml) in patients with stable versus active disease as determined by MRI (Tables 1 and 2 and Supplementary Tables 2 and 3). Stable MRI was defined as no new lesion or contrast-absorbing lesion since the last imaging, which occurred 1 year in advance of the blood sampling. Active MRI was defined as a new gadolinium-enhanced brain lesion and/or new T_2_ lesion at the time of blood sampling. Unpaired two-tailed *t*-test with Welch’s correction; *P < 0.05. (**B**) Timeline of MRI scans and blood sampling in the disease course of 40 RRMS patients used for the longitudinal assessment of sPD-L1 serum levels in respect to disease activity. (**C**) sPD-L1 serum levels in 40 RRMS patients with available MRI data 1 year (1.04 ± 0.4 years) before blood sampling, during blood sampling and 1 year after blood sampling were analysed. Lesion load volume change and white matter volume loss (WMVL) between first and second MRI (upper; 1.04 ± 0.4 years) as well as between first and third MRI (lower; 2.25 ± 0.57 years) were assessed and correlated to sPD-L1 levels analysed at the time of the second MRI. Numbers indicated R^2^ and *P*-value of linear regression analysis. For WMVL, significance levels were determined by unpaired two-tailed *t*-test with Welch’s correction; *P < 0.05.

In addition to clinical disability measures, we next aimed to establish a potential association between sPD-L1 levels and disease activity using MRI. Indeed, MS patients with a stable MRI image, defined as patients without new or contrast-enhancing lesions within 1 year prior to blood sampling, exhibited higher sPD-L1 levels as compared to patients with new or contrast-enhancing lesions (Fig. 4A, Table 2 and Supplementary Table 3). Next, we evaluated a potential correlation between sPD-L1 levels and changes in WMV or lesion size using the software package SPM and its toolboxes CAT12 and LST.^32^ To that end, we analysed changes in lesion volume and WMV within 1 year (1.04 ± 0.4 years) before blood sampling and correlated these changes to sPD-L1 levels at the time of the second MRI scan (Fig. 4B and C). While we observed no connection between changes in WMV and sPD-L1 levels (Fig. 4C, upper row left), higher sPD-L1 levels at the time of the second MRI correlated with a reduction in lesion volume (Fig. 4C, upper row right). This finding was more pronounced when we analysed changes in lesion volume and WMV over a course of 2 years (2.25 ± 0.57 years), longitudinally assessing changes between first and third MRI in respect to sPD-L1 levels taken at the time of the second MRI scan (Fig. 4A and B, lower row). Indeed, over this prolonged period, we observed higher sPD-L1 levels in those patients without WMV loss (Fig. 4C, lower row left). Similarly, higher levels of sPD-L1 correlated with reduced lesion volume changes over 2 years (Fig. 4C, lower row right). Overall, these data indicate that sPD-L1 levels correlate not only with disease disability but also MRI activity measures and may harbour a predictive potential for disease progression and activity worth evaluating in future studies.

**Table 2:** Subset of RRMS patients with available MRI imaging data

Figure	Cohorts	Females	Age	Disease duration	EDSS	Treatment
4B and C	RRMS (46)	34 (73.9%)	42.6 [36.0: 49.8]	7.5 [3.3: 9.4]	1.9 [0.0: 2.09]	37 (80.4%)

‘Females’ indicates both the absolute count and percentage of females within the group. ‘Age’, ‘Disease duration’ and ‘EDSS’ are presented as mean values, with the 25th and 75th percentiles indicated in square brackets. ‘Treatment’ represents the absolute count and percentage of patients who received treatment, while specific individual treatments as well as individual information about MRI stability, lesion load volume change and white matter volume loss (WMVL) are listed in Supplementary Tables 2 and 3. No additional noteworthy comorbidities or pharmaceutical treatments were reported among the patients.

## Discussion

Here, we report the use of in-depth immunophenotyping of MS patients and controls to investigate the association of PD-1/PD-L1 with clinical disease measures in MS and to evaluate the potential of the PD-1/PD-L1 axis as a potential target for therapeutic modulation during acute CNS inflammation.

Upregulated PD-1 levels are commonly associated with immunosuppression in physiological self-tolerance mechanisms and pathological immune exhaustion. While this has been extensively studied in the context of tumour immune escape mechanisms and chronic viral infections (reviewed in^40,41^), thorough investigation of PD-1 and particularly PD-L1 on immune cell subsets in the context of MS is limited. A previous study^42^ has, however, reported an association between decreased PD-1 activity and a progressive disease course in MS patients, which may potentially be caused by a partial failure in PD-1-mediated suppression of T cell activation.^42^ Moreover, a population-based case-control study investigating PD-1 single-nucleotide polymorphisms (SNPs) revealed that polymorphic variations affect disease progression but not development.^43^ Additional reports demonstrate that circulating CD8+ PD-1+ T cells decrease during disease remission, while CD8+ PD-1+ T cells are increased in the CSF of MS patients during relapses.^44^ The decreased expression of PD-1 by circulating CD8+cells was particularly prominent in IL10+ CD8 T cells with a regulatory phenotype in patients treated with IFN-β, supporting our observations of distinct PD-1/PD-L1 regulation by inflammatory and regulatory T cell subsets.

In agreement with the existing literature, our data identify PD-1 as significantly regulated surface marker in T cells of RRMS patients compared to controls. Subtype analysis revealed increased numbers of pro-inflammatory PD-1+ Th1, Th17, Tc1 and Tc17 T cell subsets in MS patients compared to controls, while the number of PD-1+ Treg cells was reduced. In line with previous reports describing upregulation of PD-1 as a result of T cell activation,^45^ these data demonstrate the dysregulation of PD-1 on inflammatory and regulatory T cell subsets as part of an elevated immune response in MS. Correspondingly, we observed an increase of various PD-L1^+^ cell populations in RRMS patients, collectively supporting the notion that the PD-1/PD-L1 axis presents an immune checkpoint relevant in MS pathogenesis regulated in a cell-type-specific manner.^46-48^

While the available data require additional longitudinal investigation of PD-1/PD-L1 on immune cell subsets in MS patients, previous reports suggest that this axis may be differentially regulated in distinct stages of the disease.

Indeed, a study by Javan *et al*. reported the downregulation of PD-1/PD-L1 in PBMCs of relapsing RRMS patients, highlighting the dualistic role of PD-1/PD-L1 co-stimulatory signalling in different stages of RRMS. In this context, future studies will be required to examine the disease-stage-specific role of PD-1/PD-L1 signalling, particularly in patients converting into progressive stages of MS.

The regulation of the PD-1/PD-L1 axis in MS may provide a useful tool to monitor disease activity and response to therapy in MS. In this respect, soluble PD-L1 has been found upregulated in the serum of patients with systemic lupus erythematosus,^49^ systemic sclerosis,^26^ rheumatoid arthritis,^50^ and psoriatic arthritis,^51^ while this has not been investigated in the context of MS. To our knowledge, we here describe for the first time upregulated sPD-L1 levels in sera of MS patients compared to controls. This increase in soluble PD-L1 levels negatively correlated with disease disability and activity measures, determined by EDSS score and MRI studies, supporting the hypothesis that increased PD-1/PD-L1 co-regulatory signalling in MS patients might be associated with a stable MS phenotype.^52,53^ Future studies will be needed to determine whether this correlation is due to a failure of co-regulatory mechanisms in patients with RRMS.

Several limitations of our study need to be taken into account. The number of samples analysed is limited, which compromises statistical analyses especially in patient subgroups; indeed, defining patient fractions within this limited cohort might impact the outcome of these analyses, warranting caution in drawing definitive conclusions as to specific subgroups from these results. Moreover, longitudinal analyses of individual patients have only been included to a limited extent. Yet, the focus of this study is set on the regulation of the PD-1/PD-L1 axis in MS in principle and has indeed revealed disease-specific alterations as compared to controls. Future studies should thus analyse the regulation of the PD-1/PD-L1 axis in larger patient cohorts, exploring treatment responses, investigating in-depth subgroup analyses and including longitudinal samples of stable versus relapsing and progressive MS patients. Finally, the translational and potentially therapeutic relevance of these findings needs to be addressed in future interventional studies in MS and its animal models.

In summary, we here report the dysregulation of the PD-1/PD-L1 axis in MS. This dysregulation is specific to immune cell subtypes, making PD-1 and PD-L1 candidate markers of disease in the context of MS. Further longitudinal studies will be required to determine the relevance of membrane-bound and soluble PD-L1 and PD-1 levels as putative markers of disease activity and to what extent they can be used to improve clinical decision-making.

## Supplementary Material

fcad206_Supplementary_Data

## Data Availability

Anonymized data that are not published in this article will be made available on request from any qualified investigator after the approval by the Department of Neurology of the Technical University of Munich, Germany.
