# Supplementary material for: Regulation of the programmed cell death protein 1/programmed cell death ligand 1 axis in relapsing–remitting multiple sclerosis
Source: Brain Commun. 2023 Jul 25;5(4):fcad206. doi: 10.1093/braincomms/fcad206 (PMC10411318; doi:10.1093/braincomms/fcad206)
Supplement: fcad206_Supplementary_Data [file fcad206_supplementary_data.pdf]

Supplementary Figure 1: Cluster and SAM analysis of multiparametric flow cytometry data

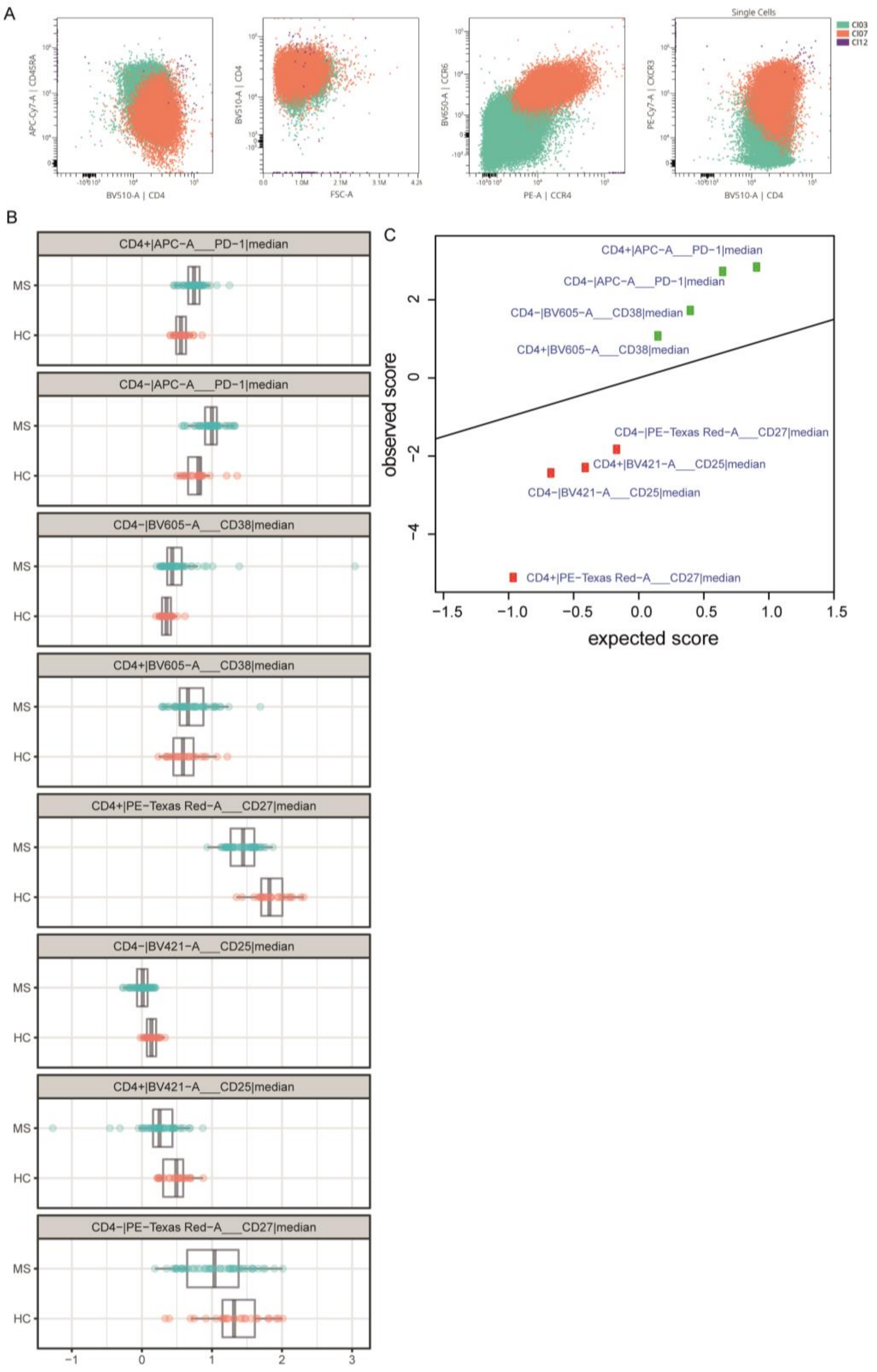

(**A**) Analysis of Cluster 3, Cluster 7 and Cluster 12 identified by FlowSOM cluster based on their expression of CD4, CD45RA, CCR6, CCR4 and CXCR3. (**B**) SAM analysis of

CD3+CD4+ and CD3+CD4- cells in healthy controls (CONT) and Multiple Sclerosis (MS) patients based on the median fluorescent intensities of activation markers PD-1, CD38, CD27, CD25. Two class unpaired analysis with 100 permutations and 0.1 FDR cutoff. Features are ranked by significance. (**C**) Significance features score plot of SAM analysis.

Supplementary Figure 2: Membranous PD-1 expression on PBMCs

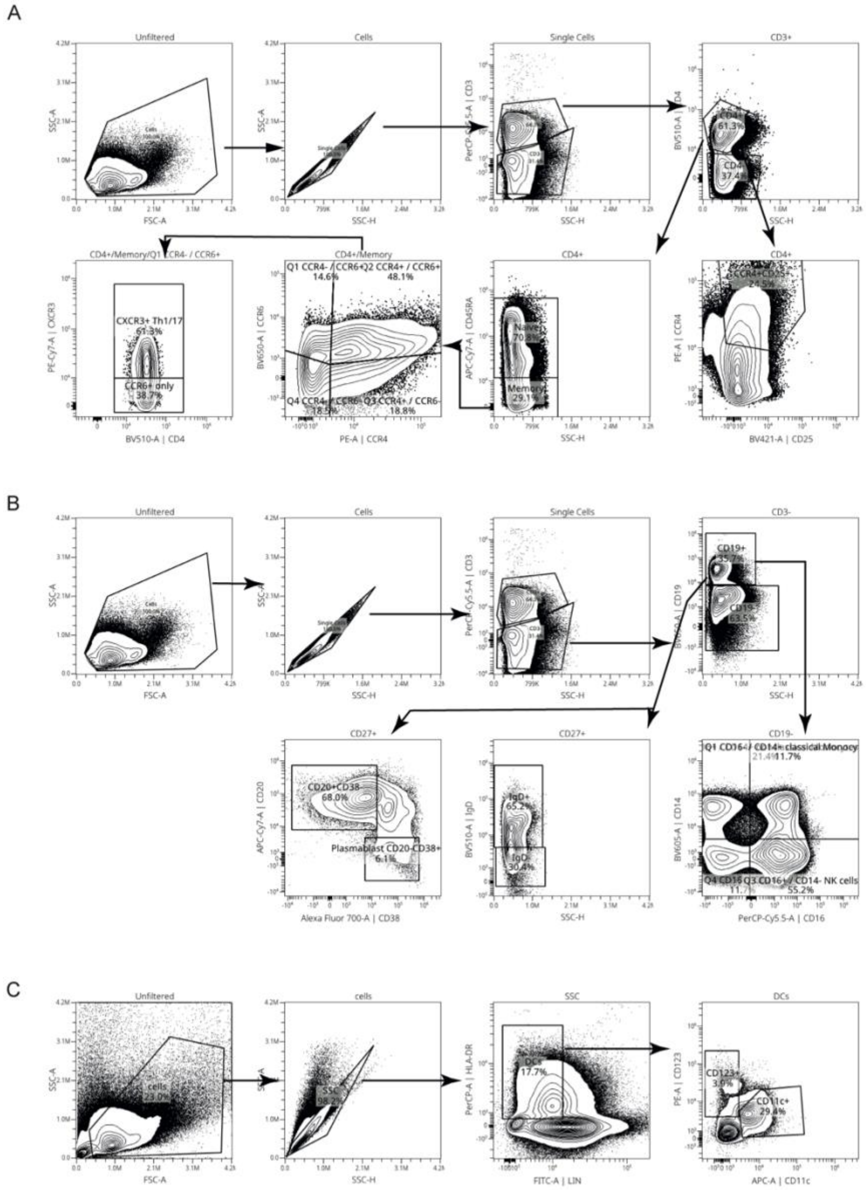

(**A**) Gating strategy for T cell panel. Following FSC-A/SSC-A discrimination, cell types were gated by positive or negative selection based on surface marker expression. CD45RA+, CCR4-, CCR6- (Th0); CD45RA-, CCR4-, CCR6- (Th2); CCR4+, CCR6+, CXCR3- (Th17), CCR4+, CCR6-, CXCR3+ (Th1). (**B**) Gating for B cell panel, monocyte subpopulations and NK cells. Following FSC-A/SSC-A discrimination, cell types were gated by positive or negative selection based on surface marker expression. CD3-, CD19+ (B cells); CD3-, CD19+, CD27- (naïve B

cells); CD3-, CD19+, CD27+ (memory B cells); CD3-, CD19+, CD27+, CD20-, CD38+ (plasmablasts); CD3-, CD19+, CD27+, IgD+ (IgD+ B cells); CD3-, CD19-, CD14+ (monocytes); CD3-, CD19-, CD14+, CD16- (classical monocytes); CD3-, CD19-, CD14+, CD16+ (non-classical monocytes), CD3-, CD19-, CD14-, CD16+ (NK cells). (**C**) Gating for dendritic cell subpopulations. Following FSC-A/SSC-A discrimination, cell types were gated by positive or negative selection based on surface marker expression. LIN-, HLA-DR+ (dendritic cells), LIN-, HLA-DR+, CD11c+ (myeloid dendritic cells), LIN-, HLA-DR+, CD11c- (plasmacytoid dendritic cells). CD = cluster of differentiation, LIN = lineage specific markers (CD3, CD14, CD16, CD19, CD20, CD56)

Supplementary Figure 3: Membranous PD-1 expression on PBMCs

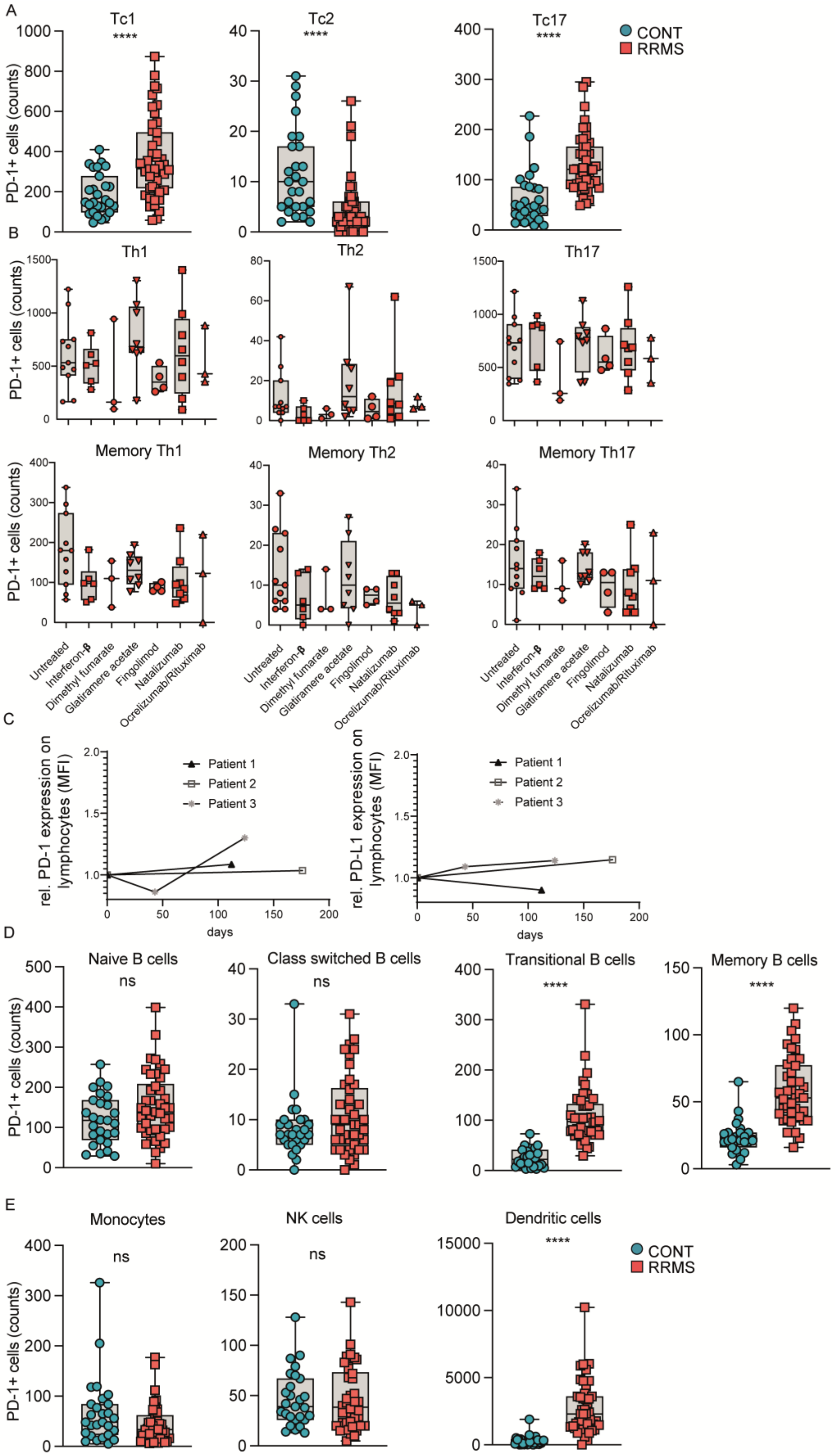

(**A**) PD-1 positive Tc1, Tc2 and Tc17 cells in controls (CONT) and Multiple Sclerosis (MS) patients. Unpaired two-tailed t-test with Welch's correction; ****P < 0.0001; Tc=T cytotoxic cells. (**B**) Detailed comparison of subtypes of T cells expressing PD-1 between non-treated and RRMS patients treated with interferon- β, dimethyl fumarate, glatiramere acetate, fingolimod, natalizumab, ocrelizumab/rituximab. Significance levels were determined by One-way ANOVA. (**C**) Relative surface expressions of PD-1 and PD-L1 on lymphocytes was measured over different time periods in three patients with RRMS. Normalization was done based on initial MFI. For patient 1, the interval between blood samples was 2 days, for patient 2 176 days, and for patient 3 124 days. (**D**) PD-1 positive naïve B cells, class switched B cells, transitional B cells, memory B cells in controls (CONT) and Multiple Sclerosis (MS) patients. Unpaired two-tailed t-test with Welch's correction; ns not significant; ****P < 0.0001. (E) Surface expression of PD-1 (counts) on monocytes, NK cells and dendritic cells in controls (CONT) and Multiple Sclerosis (MS) patients. Unpaired two-tailed t-test with Welch's correction; ns not significant; ****P < 0.0001; NK=natural killer cells

Supplementary Figure 4: Patient age, gender and disease duration do not influence sPD-L1 concentration.

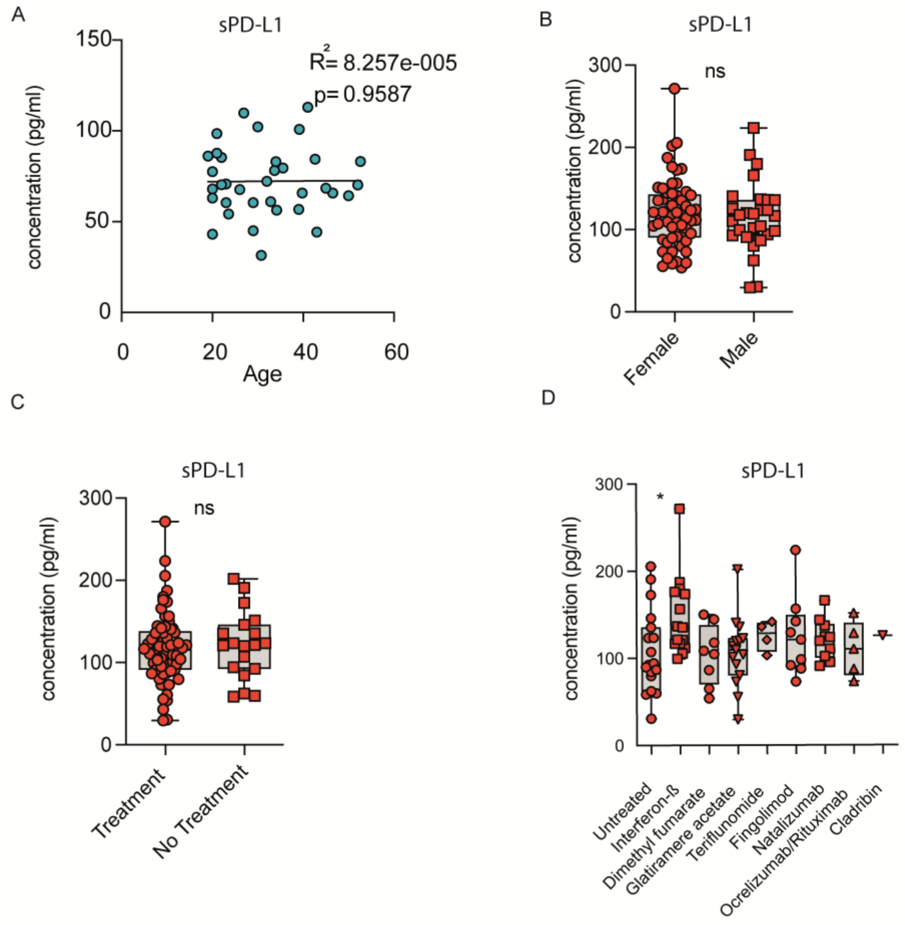

(**A**) Linear regression analysis of correlation between sPD-L1 concentration and patient age in control samples (n=36). Numbers indicate R^2 and *P* value of linear regression analysis. (**B**) Comparison of sPD-L1 concentration between female and male RRMS patients (n=84). Significance levels were determined by unpaired two-tailed t-test with Welch's correction, ns not significant. (**C**) Linear regression analysis of sPD-L1 concentration and disease duration since onset in RRMS patients (n=84) (**D**) Analysis of sPD-L1 concentration in non-treated and RRMS patients treated with interferon- β, dimethyl fumarate, glatiramere acetate, teriflunomide, fingolimod, natalizumab, ocrelizumab/rituximab, or cladribin (right, n=84). Significance levels were determined by One-way ANOVA; ns not significant; *$P < 0$ .05 (Supplementary Table 2).

Supplementary Table 1: Control patients used for the analysis of membrane-bound and soluble PD-1 / PD-L1.

ID # Controls (36)	Serum	PBMCs	Age	Sex
1	Yes	Yes	35	female
2	Yes	Yes	33	female
3	Yes	Yes	39	female
4	Yes	Yes	31	female
5	Yes	Yes	24	female
6	Yes	Yes	34	female
7	Yes	Yes	43	female
8	Yes	Yes	33	female
9	Yes	Yes	47	female
10	Yes	Yes	53	female
11	Yes	Yes	19	female
12	Yes	Yes	27	female
13	Yes	Yes	39	female
14	Yes	Yes	41	female
15	Yes	Yes	43	male
16	Yes	Yes	34	female
17	Yes	Yes	76	male
18	Yes	Yes	21	female
19	Yes	Yes	39	male
20	Yes	Yes	29	female

21	Yes	Yes	23	female
22	Yes	Yes	52	female
23	Yes	Yes	22	female
24	Yes	Yes	26	female
25	Yes	Yes	20	female
26	Yes	Yes	30	female
27	Yes	Yes	29	male
28	Yes	None	23	male
29	Yes	None	20	female
30	Yes	None	21	male
31	Yes	None	20	male
32	Yes	None	45	female
33	Yes	None	22	male
34	Yes	None	50	male
35	Yes	None	32	male
36	Yes	None	43	male

Supplementary Table 2: RRMS patients used for the analysis of membrane-bound and soluble PD-1 / PD-L1.

ID # RRMS (84)	Serum	PBMCs	Age	Sex	Duration (y)	EDSS	Treatment	Treatment history	MRI-activity	Clinical activity
1	Yes	Yes	32	female	7	3.5	Fingolimod		stable	Remission
2	Yes	Yes	28	female	1	1	Natalizumab	Interferon-β, Fingolimod	NA	Remission
3	Yes	Yes	67	female	17	3.5	Interferon-β		active	Remission
4	Yes	Yes	53	female	4	2	Teriflunomide		stable	Remission
5	Yes	Yes	45	female	3	0	Dimethyl fumarate	Interferon-β	stable	Remission
6	Yes	Yes	36	male	2	3	Fingolimod	Interferon-β	stable	Remission
7	Yes	Yes	37	female	13	2	Natalizumab	Interferon-β	stable	Remission
8	Yes	Yes	50	female	3	2.5	Fingolimod	Interferon-β. Glatiramere acetate	stable	Remission
9	Yes	Yes	49	female	7	0	Glatiramere acetate	Interferon-β	stable	Remission

10	Yes	Yes	41	female	5	0	Untreated		stable	Remission
11	Yes	Yes	38	female	3	0	Glatiramere acetate		stable	Remission
12	Yes	Yes	34	female	0	1	Glatiramere acetate	Interferon-β	active	Remission
13	Yes	Yes	39	female	0	3.5	Untreated		active	Remission
14	Yes	Yes	40	female	6	4	Dimethyl fumarate	Glatiramere acetate	stable	Remission
15	Yes	Yes	51	female	1	3.5	Natalizumab		stable	Remission
16	Yes	Yes	40	female	11	0	Interferon-β		active	Remission
17	Yes	Yes	46	female	7	2	Untreated		stable	Remission
18	Yes	Yes	47	female	4	6.5	Untreated		active	Remission
19	Yes	Yes	35	female	3	4.5	Untreated		active	Remission
20	Yes	Yes	37	female	1	2.5	Interferon-β		stable	Remission
21	Yes	Yes	63	female	13	4	Interferon-β		active	Remission
22	Yes	Yes	37	female	20	2	Fingolimod	Interferon-β, Dimethylfumarat	stable	Remission
23	Yes	Yes	40	male	5	0	Natalizumab	Dimethylfumarat	stable	Remission
24	Yes	Yes	51	male	7	6	Untreated		active	Remission
25	Yes	Yes	41	female	5	2.5	Dimethyl fumarate		NA	Remission
26	Yes	Yes	50	female	3	2.5	Glatiramere acetate		stable	Remission
27	Yes	Yes	33	male	8	2	Cladribin	Interferon-β, Dimethyl fumarate, Glatiramere acetate, Fingolimod	stable	Remission
28	Yes	Yes	42	female	23	3	Interferon-β		stable	Remission
29	Yes	Yes	34	female	8	0	Untreated	Interferon-β, Glatiramere acetate	active	Remission
30	Yes	Yes	22	female	5	1.5	Ocrelizumab	Dimethyl fumarate, Natalizumab	active	Remission
31	Yes	Yes	49	male	12	2	Interferon-β		stable	Remission
32	Yes	Yes	33	male	1	1.5	Natalizumab		NA	Remission

33	Yes	Yes	52	female	10	3.5	Fingolimod		NA	Remission
34	Yes	Yes	50	female	*25*	5.5	Interferon-β		NA	Remission
35	Yes	Yes	55	male	0	3.5	Untreated		NA	Remission
36	Yes	Yes	51	male	13	4.0	Interferon-β		NA	Remission
37	Yes	Yes	59	female	33	4.0	Untreated	Daclizumab	NA	Remission
38	Yes	Yes	45	female	21	7.0	Glatiramere acetate		NA	Remission
39	Yes	Yes	53	female	26	4.5	Untreated	Interferon-β, Dimethylfumarat	NA	Remission
40	Yes	Yes	48	male	17	3.5	Natalizumab		NA	Remission
41	Yes	Yes	58	male	12	3.5	Untreated	none	active	Remission
42	Yes	Yes	55	male	23	4.5	Glatiramere acetate		NA	Remission
43	Yes	Yes	43	male	1	4.0	Untreated		NA	Remission
44	Yes	Yes	41	female	23	4.5	Ocrelizumab	Interferon-β, Fingolimod, Natalizumab, Daclizumab	NA	Remission
45	Yes	Yes	40	male	20	4	Ocrelizumab	Glatiramere acetate, Natalizumab	stable	Remission
46	Yes	Yes	54	male	*7*	6.5	Glatiramere acetate		active	Remission
47	Yes	Yes	29	male	7	2	Glatiramere acetate		NA	Remission
48	Yes	Yes	45	male	11	2	Natalizumab	Glatiramere acetate	NA	Remission
49	Yes	Yes	47	male	14	2	Natalizumab	Interferon-β, Dimethyl fumarate	NA	Remission
50	Yes	None	38	male	8	0	Untreated		stable	Remission
51	Yes	None	44	male	0	1	Glatiramere acetate		stable	Remission
52	Yes	None	64	female	5	2	Glatiramere acetate	Interferon-β	active	Remission
53	Yes	None	55	female	8	2.5	Fingolimod	Interferon-β, Glatiramere acetate	stable	Remission

54	Yes	None	43	female	6	0	Glatiramere acetate		stable	Remission
55	Yes	None	26	female	6	0	Dimethyl fumarate	Interferon-β	stable	Remission
56	Yes	None	40	female	15	0	Dimethyl fumarate	Interferon-β	stable	Remission
57	Yes	None	56	female	15	1.5	Interferon-β		stable	Remission
58	Yes	None	43	male	9,5	0	Fingolimod	Interferon-β, Dimethyl fumarate	stable	Remission
59	Yes	None	37	female	3	0	Interferon-β		stable	Remission
60	Yes	None	34	female	9	0	Interferon-β		NA	Remission
61	Yes	None	33	female	3	0	Dimethyl fumarate		stable	Remission
62	Yes	None	51	female	3	1	Dimethyl fumarate		active	Remission
63	Yes	None	37	female	8	2.5	Ocrelizumab	Natalizumab	stable	Remission
64	Yes	None	33	male	6	2	Glatiramere acetate		NA	Remission
65	Yes	None	36	female	2	2	Interferon-β		NA	Remission
66	Yes	None	28	female	2	2	Fingolimod	Interferon-β, Dimethyl fumarate	NA	Remission
67	Yes	None	23	female	0	1.5	Untreated		NA	Remission
68	Yes	None	36	female	1	0	Untreated		stable	Remission
69	Yes	None	32	male	7	1	Teriflunomide	Glatiramere acetate	stable	Remission
70	Yes	None	36	male	1	1	Interferon-β		stable	Remission
71	Yes	None	58	male	10	2	Dimethyl fumarate		NA	Remission
72	Yes	None	37	male	13	1	Glatiramere acetate		active	Remission
73	Yes	None	44	female	0	2	Untreated		NA	Remission
74	Yes	None	58	female	2	1.5	Untreated		NA	Remission
75	Yes	None	47	male	6	1.5	Glatiramere acetate	Interferon-β, Dimethyl fumarate	NA	Remission

76	Yes	None	32	male	9	1	Natalizumab	Glatiramere acetate	stable	Remission
77	Yes	None	47	female	2	3	Natalizumab	Glatiramere acetate	stable	Remission
78	Yes	None	37	female	2	0	Interferon-β		NA	Remission
79	Yes	None	41	female	6	2.5	Interferon-β		NA	Remission
80	Yes	None	38	female	3	1	Untreated	Interferon-β	stable	Remission
81	Yes	None	42	female	0	1	Teriflunomide		stable	Remission
82	Yes	None	34	female	6	1.5	Fingolimod		stable	Remission
83	Yes	None	38	female	20	6	Teriflunomide	Interferon-β, Fingolimod	NA	Remission
84	Yes	None	42	female	13	2	Untreated		stable	Remission

NA: not available; Disease: disease duration

Supplementary Table 3: Subset of RRMS patients (Table 1) with available MRI imaging data.

ID # RRMS (46)	Age	Sex	Duration (y)	EDSS	Treatment	1.MRI	2. MRI (blood)	3. MRI	Delta between 2.	Delta between 3.
1	32	female	7	3.5	Fingolimod	2015-03-09	2016-08-30	2019-10-29	1 year	4 years
Lesion volume						2,248	1,99	1,683	-11,48%	-25,13%
WMV						428,09	432,9	449,2	1,13%	4,94%
3	67	female	17	3.5	Interferon-β	2014-03-03	2015-01-23	2018-01-10	1 year	4 years
Lesion volume						47,838	50,835	42,844	6,26%	-10,44%
WMV						450,1	452,0	462,0	0,42%	2,63%
4	53	female	4	2	Teriflunomide	2017-03-23	2018-03-21	2019-03-28	1 year	2 years
Lesion volume						3,696	1,889	2,3	-48,89%	-37,77%
WMV						359,5	372,6	363,4	3,63%	1,08%
5	45	female	3	0	Dimethyl fumarate	2017-05-15	2018-04-24	2019-04-30	1 year	2 years

Lesion volume						14,061	12,199	13,656	-13,24%	-2,88%
WMV						491,2	510,7	503,2	3,96%	2,45%
6	36	male	2	3	Fingolimod	2017-04-27	2019-04-15	2020-03-11	1 year	3 years
Lesion volume						9,522	9,307	9,084	-2,26%	-4,60%
WMV						505,5	523,7	521,9	3,60%	3,23%
7	37	female	13	2	Natalizumab	2017-06-13	2018-04-04	2019-04-15	1 year	2 years
Lesion volume						31,9	30,3	31,2	-4,90%	-2,02%
WMV						414,5	438,4	437,5	5,78%	5,56%
8	50	female	3	2.5	Fingolimod	2017-05-03	2018-03-21	2019-03-22	1 year	2 years
Lesion volume						5,45	4,435	5,682	-18,62%	4,26%
WMV						433,1	451,9	447,8	4,33%	3,39%
9	49	female	7	0	Glatiramere acetate	2017-04-13	2018-04-12	2019-04-18	1 year	2 years
Lesion volume						1,101	0,69	0,824	-37,33%	-25,16%
WMV						420,3	436,2	432,6	3,80%	2,93%
10	41	female	5	0	Untreated	2016-05-23	2018-04-17	2020-11-27	1 year	4 years
Lesion volume						0,46	0,09	0,148	-80,43%	-67,83%
WMV						519,9751587	540,5634766	530,5006714	3,96%	2,02%
11	38	female	3	0	Glatiramere acetate	2017-04-20	2018-04-19	2019-04-17	1 year	2 years
Lesion volume						3,363	2,634	2,446	-21,68%	-27,27%
WMV						398,4428101	417,7327881	418,5944519	4,84%	5,06%
12	34	female	0	1	Glatiramere acetate	2017-04-20	2018-04-19	2019-04-18	1 year	2 years

Lesion volume						4,549	2,692	3,597	-40,82%	-20,93%
WMV						505,546 0815	518,251 8311	522,202 0874	2,51%	3,29%
13	39	female	0	3.5	Untreated	2014-04-29	2015-04-30	2016-04-26	1 year	2 years
Lesion volume						1,422	1,535	1,205	7,95%	-15,26%
WMV						485,191 3147	478,676 6968	473,232 605	-1,34%	-2,46%
14	40	female	6	4	Dimethyl fumarate	2013-08-23	2014-08-26	2015-08-26	1 year	2 years
Lesion volume						4,918	6,573	6,922	33,65%	40,75%
WMV						406,498 1689	404,568 6951	399,942 5354	-0,47%	-1,61%
15	51	female	1	3.5	Natalizumab	2014-09-15	2016-09-12	2017-10-30	1 year	3 years
Lesion volume						17,595	21,366	16,234	21,43%	-7,74%
WMV						513,489 3799	506,353 9734	521,153 8696	-1,39%	1,49%
16	40	female	11	0	Interferon-β	2017-12-11	2018-06-18	2019-11-11	1 year	2 years
Lesion volume						0,11	0,149	0,244	35,45%	121,8%
WMV						475,812 6526	471,123 9319	471,878 3569	-0,99%	-0,83%
17	46	female	7	2	Untreated	2017-05-10	2018-04-11	2019-04-08	1 year	2 years
Lesion volume						6,607	4,262	4,584	-35,49%	-30,62%
WMV						532,578 3081	554,979 187	552,043 0908	4,21%	3,65%
18	47	female	4	6.5	Untreated	2014-04-07	2015-04-13	2016-04-25	1 year	2 years

Lesion volume						0,787	0,77	0,774	-2,16%	-1,65%
WMV						431,066864	424,4631653	427,1767578	-1,53%	-0,90%
21	63	female	13	4	Interferon-β	2016-09-12	2018-03-08	2019-10-31	1 year	3 years
Lesion volume						11,52	10,787	11,098	-6,36%	-3,66%
WMV						409,7063904	424,7710876	424,0983887	3,68%	3,51%
22	37	female	20	2	Fingolimod	2017-04-13	2018-04-26	2019-04-23	1 year	2 years
Lesion volume						21,252	33,274	34,873	56,57%	64,09%
WMV						426,7368164	426,1116638	411,8479919	-0,15%	-3,49%
23	40	male	5	0	Natalizumab	2017-04-24	2018-04-23	2019-05-21	1 year	2 years
Lesion volume						3,268	1,978	1,47	-39,47%	-55,02%
WMV						533,7562256	553,8724365	552,3010864	3,77%	3,47%
24	51	male	7	6	Untreated	2014-04-24	2015-04-23	2016-04-27	1 year	2 years
Lesion volume						2,737	2,7	3,05	-1,35%	11,44%
WMV						496,7282104	498,3797913	496,1959534	0,33%	-0,11%
26	50	female	3	2.5	Glatiramere acetate	2017-06-12	2018-04-16	2019-07-05	1 year	2 years
Lesion volume						1,734	1,307	1,319	-24,63%	-23,93%
WMV						455,6099854	468,7895813	468,4484253	2,89%	2,82%
27	33	male	8	2	Cladribin	2017-03-29	2018-03-26	2019-06-17	1 year	2 years

Lesion volume						26,243	24,418	26,537	-6,95%	1,12%
WMV						427,618 4387	465,778 2898	465,556 5796	8,92%	8,87%
28	42	female	23	3	Interferon-β	2017-06-06	2018-04-16	2019-04-08	1 year	2 years
Lesion volume						0,629	0,275	0,408	-56,28%	-35,14%
WMV						494,392 4866	515,499 3286	508,189 5447	4,27%	2,79%
29	34	female	8	0	Untreated	2017-06-01	2018-04-16	2019-12-10	1 year	2 years
Lesion volume						0,575	0,379	0,551	-34,09%	-4,17%
WMV						512,289 4287	530,144 9585	532,526 001	3,49%	3,95%
30	22	female	5	1.5	Ocrelizumab	2017-07-03	2018-01-30	2019-03-12	1 year	2 years
Lesion volume						1,381	0,439	0,906	-68,21%	-34,40%
WMV						407,237 6404	436,721 344	435,825 0732	7,24%	7,02%
31	49	male	12	2	Interferon-β	2017-04-19	2018-02-01	2019-05-28	1 year	2 years
Lesion volume						0,471	0,218	0,179	-53,72%	-62,00%
WMV						556,175 7813	571,498 291	569,443 1763	2,75%	2,39%
45	40	male	20	4	Ocrelizumab	2015-08-19	2018-05-18	2019-05-08	1 year	4 years
Lesion volume						34,392	37,832	38,701	10,00%	12,53%
WMV						504,735 8398	507,849 884	508,183 7769	0,62%	0,68%
46	54	male	7	6.5	Glatiramere acetate	2017-05-04	2018-04-18	2019-07-29	1 year	2 years

Lesion volume						2,871	2,136	4,086	-25,60%	42,32%
WMV						498,039 5203	513,880 6152	505,150 238	3,18%	1,43%
50	38	male	8	0	Untreated	2017-05-18	2018-03-21	2019-03-05	1 year	2 years
Lesion volume						3,808	3,468	4,003	-8,93%	5,12%
WMV						564,271 0571	577,970 1538	571,593 3228	2,43%	1,30%
52	64	female	5	2	Glatiramere acetate	2017-04-12	2018-04-05	2019-03-19	1 year	2 years
Lesion volume						0,22	0,195	0,212	-11,36%	-3,64%
WMV						452,350 647	473,180 2063	469,313 2324	4,60%	3,75%
53	55	female	8	2.5	Fingolimod	2017-06-06	2018-04-09	2019-10-04	1 year	2 years
Lesion volume						8,5	6,535	7,147	-23,12%	-15,92%
WMV						531,983 1543	554,390 9302	548,707 8857	4,21%	3,14%
54	43	female	6	0	Glatiramere acetate	2017-05-10	2018-04-12	2019-06-13	1 year	2 years
Lesion volume						1,715	0,791	1,105	-53,88%	-35,57%
WMV						435,962 0056	465,223 0835	456,397 2473	6,71%	4,69%
55	26	female	6	0	Dimethyl fumarate	2017-06-08	2018-04-18	2019-03-28	1 year	2 years
Lesion volume						0,924	0,367	0,498	-60,28%	-46,10%
WMV						513,546 9971	540,766 2354	532,520 752	5,30%	3,69%
56	40	female	15	0	Dimethyl fumarate	2017-03-23	2018-04-19	2019-09-19		

Lesion volume						0,619	0,286	0,485	-53,80%	-21,65%
WMV						455,295 7764	472,950 3784	467,081 4209	3,88%	2,59%
57	56	female	15	1.5	Interferon-β	2017-06-12	2018-04-23	2019-04-29	1 year	2 years
Lesion volume						8,818	6,037	7,593	-31,54%	-13,89%
WMV						438,125 2441	462,196 9604	463,551 6357	5,49%	5,80%
58	43	male	9,5	0	Fingolimod	2017-03-23	2018-04-26	2019-03-28	1 year	2 years
Lesion volume						4,713	3,307	3,535	-29,83%	-24,99%
WMV						555,635 437	579,194 6411	574,842 8345	4,24%	3,46%
61	33	female	3	0	Dimethyl fumarate	2017-05-23	2018-04-10	2019-03-06	1 year	2 years
Lesion volume						10,636	8,98	9,1	-15,57%	-14,44%
WMV						486,332 8552	505,263 8855	506,255 9814	3,89%	4,10%
62	51	female	3	1	Dimethyl fumarate	2017-05-11	2018-04-18	2019-05-20	1 year	2 years
Lesion volume						4,854	4,09	5,305	-15,74%	9,29%
WMV						478,490 6006	496,296 7834	491,857 2693	3,72%	2,79%
63	37	female	8	2.5	Ocrelizumab	2017-08-16	2018-05-29	2019-08-06	1 year	2 years
Lesion volume						9,662	8,708	9,034	-9,87%	-6,50%
WMV						486,562 4695	503,475 3113	500,576 8433	3,48%	2,88%
68	36	female	1	0	Untreated	2017-03-31	2018-04-03	2019-03-20	1 year	2 years

Lesion volume						1,705	0,932	1,101	-45,34%	-35,43%
WMV						500,825 1648	515,845 1538	514,310 0586	3,00%	2,69%
69	32	male	7	1	Teriflunomide	2017-04-21	2018-04-09	2019-04-18	1 year	2 years
Lesion volume						2,928	1,469	2,236	-49,83%	-23,63%
WMV						560,647 7051	579,100 0366	576,823 1201	3,29%	2,89%
70	36	male	1	1	Interferon-β	2017-04-04	2018-04-25	2019-08-29	1 year	2 years
Lesion volume						7,518	5,542	6,836	-26,28%	-9,07%
WMV						480,070 8923	501,844 4519	500,642 5171	4,54%	4,29%
76	32	male	9	1	Natalizumab	2017-04-06	2018-04-09	2019-04-08	1 year	2 years
Lesion volume						3,39	2,031	2,568	-40,09%	-24,25%
WMV						489,529 3274	512,486 5723	504,738 3118	4,69%	3,11%
82	34	female	6	1.5	Fingolimod	2017-04-13	2018-04-12	2019-04-23	1 year	2 years
Lesion volume						5,897	5,595	6,053	-5,12%	2,65%
WMV						461,804 3213	468,259 7351	466,918 4875	1,40%	1,11%
84	42	female	13	2	Untreated	2017-04-27	2018-04-26	2019-04-29	1 year	2 years
Lesion volume						1,218	0,978	1,068	-19,70%	-12,32%
WMV						500,105 3162	518,604 5532	517,329 9561	3,70%	3,44%

Note: lesion volume in ml; WMV: white matter volume (in ml); Disease: disease duration, Delta between 2.: Difference between the 1st and 2nd MRI; Delta between 3.: Difference between the 1st and 3rd MRI.
